# Diagnosis and treatment of vitamin B12 deficiency in children

**DOI:** 10.3389/fnut.2026.1883501

**Published:** 2026-07-29

**Authors:** Purva Kanvinde, Ritika Khurana, Sangeeta Mudaliar

**Affiliations:** Department of Paediatric Hematology-Oncology, Bai Jerbai Wadia Hospital for Children, Mumbai, India

**Keywords:** holotranscobalamin, inherited vitamin B12 disorders, methylmalonic acid levels, treatment of vitamin B12 deficiency, vitamin B12 deficiency

## Abstract

Vitamin B12 deficiency is a common yet frequently underrecognized nutritional disorder with important public health implications, particularly in low- and middle-income countries where maternal deficiency, vegetarian dietary practices, and food insecurity are prevalent. In children, deficiency may lead to irreversible neurodevelopmental impairment, growth failure, and hematological abnormalities, making early recognition essential. The clinical spectrum ranges from asymptomatic biochemical deficiency and isolated macrocytic anemia to developmental regression, seizures, movement disorders, peripheral neuropathy, and pancytopenia, with neurological manifestations often preceding hematological changes. Diagnosis requires integration of clinical features with laboratory evaluation, including complete blood count, peripheral smear, serum vitamin B12 estimation, and functional biomarkers such as methylmalonic acid and homocysteine, which improve diagnostic accuracy in borderline or atypical cases. Molecular testing has expanded the identification of inherited disorders affecting vitamin B12 absorption and metabolism, enabling timely lifelong management. Prompt replacement therapy is the cornerstone of treatment and should not be delayed when clinical suspicion is high. Oral supplementation is effective for most dietary deficiencies, whereas parenteral therapy is indicated for severe anemia, neurological involvement, malabsorption, or inherited cobalamin disorders. Early treatment results in rapid hematological recovery, but delayed intervention may leave permanent neurological sequelae despite biochemical correction. Beyond individual patient care, strengthening maternal nutrition, targeted screening of high-risk populations, food fortification, and public awareness are critical strategies to reduce the burden of vitamin B12 deficiency and prevent avoidable disability. This review summarizes current evidence on the diagnosis and treatment of pediatric vitamin B12 deficiency and highlights practical approaches for improving clinical outcomes.

## 1 Introduction

Vitamin B12 (cobalamin) is a water-soluble vitamin which acts as a cofactor for methionine synthase and methylmalonyl-CoA mutase, enzymes involved in homocysteine remethylation and mitochondrial fatty acid metabolism (1, 2). Deficiency results in impaired DNA synthesis, ineffective erythropoiesis, accumulation of methylmalonic acid (MMA) and homocysteine, and progressive neurological dysfunction.

The spectrum of Vitamin B12 deficiency ranges from asymptomatic biochemical deficiency to severe pancytopenia, developmental regression, seizures, movement disorders, and irreversible neurocognitive impairment (1, 2). Although megaloblastic anemia remains a classic presentation, many children may present with a normocytic anemia or isolated neurological symptoms. Delayed recognition continues to be a major challenge.

### 1.1 Prevalence

Vitamin B12 deficiency is widely prevalent yet underrecognized nutritional disorder among Indian children. The Comprehensive National Nutrition Survey (CNNS) reported deficiency in 13.8% of preschool children, 17.3% of school-age children, and 31.0% of adolescents, indicating a substantial public health burden (3). In hospital-based studies on patients attending a tertiary care center in New Delhi, vitamin B12 deficiency was seen in 37.6% of apparently healthy infants and toddlers, while Azad et al. reported deficiency in 22% of infants with an association with neurodevelopmental delay (4, 5).

### 1.2 Physiology and metabolism of vitamin B12

Vitamin B12 is obtained exclusively from animal-derived foods such as meat, eggs, milk, and fish (3). It cannot be synthesized in Human body; therefore, adequate dietary intake is essential. After ingestion, cobalamin binds salivary haptocorrin in the stomach. In the duodenum, pancreatic enzymes release vitamin B12, allowing binding to intrinsic factor (IF), secreted by gastric parietal cells. The IF–B12 complex is absorbed in the terminal ileum through cubilin-mediated uptake.

Once absorbed, cobalamin binds transcobalamin II for systemic transport. Within cells, vitamin B12 is converted into two active forms:

1. Methylcobalamin, required for methionine synthase activity.
2. Adenosylcobalamin, required for methylmalonyl-CoA mutase activity.

Disruption at any step—including intake, absorption, transport, or intracellular metabolism—can result in deficiency.

## 2 Etiology of vitamin B12 deficiency

Deficiency can arise due to acquired causes like insufficient intake, increased requirement, inadequate absorption, increased consumption due to infections or drug intake etc (5, 6, 22, 29). Acquired causes are listed in Table 1.

**Table 1:** Vitamin B12 deficiency (acquired causes).

Category	Phase of vitamin B12 metabolism affected	Specific cause/disorder	Mechanism	Typical clinical setting
Acquired causes	Inadequate intake	Exclusive breastfeeding by a vitamin B12-deficient mother	Low maternal stores lead to reduced B12 content in breast milk	Most common cause in infants, especially between 4–12 months
		Strict vegetarian or vegan diet	Absence of animal-derived foods	Older children and adolescents
		Severe malnutrition or food insecurity	Chronic inadequate intake	Low-resource settings
	Gastric phase	Autoimmune gastritis/ pernicious anemia	Autoantibodies against intrinsic factor or parietal cells reduce intrinsic factor production	Rare in children, more common in adolescents
		Long-term proton pump inhibitor (PPI) therapy	Reduced gastric acid impairs release of B12 from food proteins	Children receiving prolonged acid suppression
		Gastric surgery (gastrectomy, bariatric surgery)	Loss of intrinsic factor-producing cells	Rare in children
	Pancreatic phase	Pancreatic insufficiency (e.g., cystic fibrosis, chronic pancreatitis)	Failure to degrade haptocorrin prevents transfer of B12 to intrinsic factor	Children with exocrine pancreatic disease
	Ileal absorption phase	Crohn disease involving terminal ileum	Reduced ileal absorption of IF-B12 complex	Adolescents with inflammatory bowel disease
		Ileal resection	Loss of absorption site	Post-surgical patients
		Celiac disease (occasionally)	Secondary malabsorption due to mucosal injury	May coexist with other nutritional deficiencies
		Small intestinal bacterial overgrowth (SIBO)	Bacterial consumption of vitamin B12	Chronic gastrointestinal disorders
		Fish tapeworm (*Diphyllobothrium latum*) infestation	Parasite competes for dietary vitamin B12	Endemic regions or raw fish consumption
		HIV enteropathy or chronic intestinal disease	Malabsorption	Rare in pediatric setting
	Transport/utilization	Nitrous oxide exposure	Oxidizes active cobalamin, causing functional deficiency	Repeated anesthesia or recreational exposure in adolescents
		Certain medications (metformin, colchicine, neomycin)	Interfere with absorption or metabolism	Long-term therapy
	Increased requirement	Prematurity and low birth weight	Limited hepatic stores at birth	Neonates and young infants
		Rapid growth	Increased metabolic demand	Infancy and adolescence

### 2.1 Presentation

#### 2.1.1 Hematological manifestations

The most common hematological manifestation is macrocytic or megaloblastic anemia (7, 8). Leukopenia and thrombocytopenia are generally less common than anemia but may coexist with it, resulting in pancytopenia in advanced disease. In a retrospective pediatric study by Aygün et al., anemia was identified in only 18.2% of vitamin B12-deficient children, while neutropenia and thrombocytopenia were observed in 8.4 and 0.8% of cases, respectively, emphasizing that normal blood counts do not exclude deficiency (9).

Pseudo- thrombotic thrombocytopenic purpura has been reported in approximately 2–3% of patients with severe cobalamin deficiency and characterized by microangiopathic hemolytic anemia, schistocytosis, thrombocytopenia, markedly elevated LDH, and neurological symptoms that mimic true thrombotic thrombocytopenic purpura. Unlike TTP, ADAMTS13 activity is typically preserved, and patients respond dramatically to vitamin B12 replacement (10).

Although megaloblastic anemia remains the commonest hematological manifestation, increasing evidence suggests that neurological or systemic symptoms may precede hematological abnormalities, highlighting the importance of biochemical evaluation even in patients with normal hemoglobin and mean corpuscular volume.

#### 2.1.2 Neurological manifestations

A prospective pediatric study reported neurological manifestations in 65% of vitamin B12-deficient children, with developmental delay occurring in 60%, hypotonia in 38.7%, exaggerated reflexes in 31.2%, and abnormal movements in 20% (11).

##### 2.1.2.1 Infancy

Neurological involvement is particularly severe during infancy because of rapid brain development and active myelination. Common manifestations include developmental delay or regression, hypotonia, irritability, feeding difficulties (suck and swallow coordination), apathy, tremors, seizures, delayed milestones, poor social interaction and failure to thrive (12).

Infantile Tremor Syndrome (ITS) is now widely regarded as part of the spectrum of severe vitamin B12 deficiency in many populations, particularly among exclusively breastfed infants of vitamin B12-deficient mothers (12). It is characterized by developmental regression, coarse tremors, hypotonia, hyperpigmentation, sparse hair, and anemia. Tremors may involve the face, tongue, and limbs and can occasionally emerge or transiently worsen after initiation of vitamin B12 replacement. Although early treatment often results in rapid improvement in alertness and motor function; persistent deficits in cognition, language, executive function, and motor development have been documented despite biochemical correction.

In a recent follow-up study of 35 children with ITS, Gupta et al. reported that only 26% achieved an average social quotient, whereas 51% had borderline disability and 23% had mild-to-severe intellectual disability after a mean follow-up of 16 months, despite marked clinical improvement during the acute phase (13). Neuroimaging in severe cases may reveal cerebral atrophy or delayed myelination, changes that may partially reverse following timely treatment (14).

##### 2.1.2.2 Toddlers and young children

In toddlers and preschool children, manifestations include delayed speech, cognitive impairment, developmental regression, gait disturbances, hypotonia, seizures, behavioral abnormalities, learning difficulties, and feeding problems. Dysphagia may persist or newly manifest because of impaired neuromuscular coordination (15). Developmental delay remains the predominant neurological feature in this age group.

##### 2.1.2.3 Adolescents

Neurological manifestations in adolescents may occur in the absence of anemia or macrocytosis and more commonly involve the peripheral and central nervous systems. Clinical manifestations include paresthesias, numbness, peripheral neuropathy, gait instability, weakness, impaired vibration and proprioception, cognitive dysfunction, depression, irritability, memory impairment, and rarely psychosis (14). Subacute combined degeneration of the spinal cord may develop in prolonged deficiency. Bulbar involvement may occasionally produce dysphagia or dysarthria, although these are uncommon presentations (14).

#### 2.1.3 Other manifestations

Cutaneous manifestations are particularly frequent in children and include knuckle hyperpigmentation, generalized hyperpigmentation, sparse or depigmented hair, glossitis, angular cheilitis, and oral mucosal pigmentation. A prospective study reported skin and knuckle hyperpigmentation in 85% of children, sparse hair in 70%, and chubby face in 42.5% (14).

Growth failure, anorexia, poor weight gain, fatigue, recurrent infections, and failure to thrive are also common presentations (7). Gastrointestinal manifestations such as glossitis, stomatitis, diarrhea, constipation, and feeding intolerance may occur, while cardiovascular symptoms including palpitations and syncope are generally secondary to severe anemia rather than direct cobalamin deficiency.

Overall:

- Hematological abnormalities remain the traditional hallmark of vitamin B12 deficiency.
- Neurological and cutaneous manifestations—particularly in infants and young children—may predominate and often precede changes in blood counts.
- Early recognition and prompt treatment are essential to prevent irreversible neurological injury.

### 2.2 When to suspect inherited vitamin B12 deficiency

Certain indicators clinical and laboratory features can raise suspicion for inherited Vitamin B12 deficiency. Newborn screening tools may identify elevated propionylcarnitine or MMA levels (16). Early presentation in infancy or childhood with adequate nutrition history, recurrent or persistent deficiency despite supplementation need further evaluation. Abnormal triple test results such as near normal Vitamin B12 levels but elevated Homocysteine and MMA can be seen in certain inherited Vitamin B12 deficiency syndromes. Hematological abnormalities may be absent, delaying diagnosis (17).

Other system involvement in the form of disproportionate neurological manifestations related to anemia, accompanying proteinuria (as in Imerslund- Gräsbeck syndrome), pulmonary hypertension, hearing defects or thrombotic microangiopathy should undergo genetic evaluation (5, 18–20).

Table 2 describes inherited vitamin B12 disorders, clinical cues to diagnosis and suggested diagnostic approach (5, 17–20, 43).

**Table 2:** Common inherited vitamin B12 deficiency disorders and suggested diagnostic approach.

Disorder	Gene(s)	Typical age at presentation	Key clinical features	Biochemical profile	Diagnostic clues/confirmatory tests
Intrinsic factor deficiency	GIF	Infancy to childhood	Megaloblastic anemia, failure to thrive, recurrent infections, glossitis	↓ Vitamin B12, ↑ MMA, ↑ homocysteine	Normal diet, absent intrinsic factor; confirm by genetic testing
Imerslund–Gräsbeck syndrome	CUBN, AMN	Childhood	Megaloblastic anemia, failure to thrive, persistent proteinuria, neurological symptoms	↓ Vitamin B12, ↑ MMA, ↑ homocysteine	Characteristic low-grade proteinuria with normal renal function; genetic confirmation
Transcobalamin II deficiency	TCN2	Neonatal period or infancy	Severe megaloblastic anemia, diarrhea, recurrent infections, immunodeficiency, developmental delay	Low intracellular B12 despite variable serum B12, ↑ MMA, ↑ homocysteine	Consider when severe deficiency occurs in early infancy; molecular diagnosis
cblC disease (combined methylmalonic acidemia and homocystinuria)	MMACHC	Neonatal, infantile, or late-onset adulthood	Developmental delay, hypotonia, seizures, retinopathy, psychiatric symptoms, myelopathy, thromboembolism, thrombotic microangiopathy	↑ MMA, ↑ homocysteine, often ↓ methionine	Most common intracellular cobalamin disorder; confirm by genetic testing
cblD, cblF, cblJ and related combined defects	MMADHC, LMBRD1, ABCD4	Infancy or childhood (occasionally later)	Variable neurological impairment, developmental delay, anemia	↑ MMA and ↑ homocysteine	Molecular diagnosis following biochemical screening
Isolated methylmalonic acidemia (cblA, cblB, MUT)	MMAA, MMAB, MUT	Neonatal or infancy	Metabolic acidosis, vomiting, developmental delay, recurrent metabolic crises	↑ MMA with normal or minimally elevated homocysteine	Organic acids, acylcarnitine profile, genetic testing
Isolated remethylation defects (cblE, cblG)	MTRR, MTR	Childhood to adulthood	Megaloblastic anemia, cognitive impairment, neuropathy, psychiatric symptoms	↑ Homocysteine, normal MMA, ↓ methionine	Plasma amino acids and molecular testing

### 2.3 Differential diagnosis

Other causes of anemia like folate deficiency may mimic Vitamin B12 deficiency. Pancytopenia due to inherited bone marrow syndromes, leukemia, myelodysplastic syndrome etc. should be ruled out.

Table 3 enlists the common differential diagnosis, along with the clinical symptoms and laboratory findings which help in differentiating them from vitamin B12 deficiency.

**Table 3:** Differential diagnosis of vitamin B12 deficiency.

Sr. No	Condition	Clinical differences from vitamin B12	Laboratory differences from vitamin B12
1	Aplastic anemia	Bleeding manifestations, infections	Normal LDH and bilirubin, bone marrow suggestive of aplasia
2	Leukemia	Organomegaly, bone pains, bleeding manifestations	CBC may show normocytic anemia and normal RDW, Peripheral smear and bone marrow will show presence of blasts
3	Myelodysplastic syndrome	Organomegaly, transfusion requirement	Peripheral smear and bone marrow may show dysplasia in cell lines and cytogenetic abnormalities
4	Inherited bone marrow failures	Dysmorphism on clinical examination	Normal RDW on CBC, Normal LDH and bilirubin, bone marrow suggestive of aplasia
5	Iron deficiency anemia	No knuckle hyperpigmentation	Microcytic anemia and Iron studies suggestive of IDA
6	Folate Deficiency	Usually no neurological symptoms	Serum and RBC folate levels maybe low with normal MMA and high homocysteine levels
7	Thiamine Responsive Megaloblastic anemia	Presence of sensorineural hearing loss, diabetes mellitus and variable other system involvement like eye abnormalities and cardiac Response to Thiamine and not vitamin B12	Presence of ringed sideroblasts on bone marrow examination and vitamin B12 levels
8	Orotic Aciduria	Developmental delay	Elevated urinary orotic acid, normal Vitamin B12, MMA and Hcy levels
9	Lesch Nyhan syndrome	Compulsive mutilating behavior, severe neurological and motor delay	Extremely high uric acid levels, enzyme assay

### 2.4 Diagnosis

The diagnosis of vitamin B12 deficiency requires integration of clinical assessment with hematological and biochemical investigations.

Complete blood count and peripheral blood smear may show macrocytic anemia, although it is not a universal finding, especially with co-existing iron deficiency. Mean Corpuscular Volume should be interpreted as per age defined cut-offs (21). Elevated red cell distribution width (RDW) may precede overt macrocytosis. Additionally, low reticulocyte count and newer parameters such as reduced reticulocyte hemoglobin content (CHr or Ret-He) reflect impaired red cell production. The peripheral blood smear typically shows macro-ovalocytes and hypersegmented neutrophils (presence of one or more six-lobed neutrophils or at least five neutrophils with five well-separated lobes among 100 segmented neutrophils) (22). Supportive evidence includes biochemical markers of ineffective erythropoiesis such as markedly elevated lactate dehydrogenase (LDH) and indirect hyperbilirubinemia.

Measurement of serum vitamin B12 and folate constitutes the first-line biochemical investigation.

Vitamin B12 measurement technique has evolved from radioimmunoassay (RIA) to much safer automated chemiluminescence immunoassays (CLIA), the current laboratory standard owing to their speed and accessibility (23–25). Reference laboratories and research applications use more specialized methods such as high performance liquid chromatography (HPLC) and liquid chromatography- tandem mass spectrometry (LC–MS/MS) which can differentiate cobalamin forms (23–25).

Serum vitamin B12 concentrations below 200 pg./mL support a diagnosis of deficiency, values between 200 and 300 pg./mL are considered borderline and require further evaluation with functional biomarkers, MMA and homocysteine (7, 16, 21). Vitamin B12 may be falsely elevated in inflammatory disorders, liver disease, malignancy, pregnancy, folate deficiency, and certain medications. Folate deficiency may coexist with vitamin B12 deficiency and influence interpretation of homocysteine levels; therefore, simultaneous assessment of serum folate is recommended. Serum folate concentrations below 4 ng/mL and red blood cell folate levels below 100 ng/mL are suggestive of folate deficiency (16, 21).

Elevated MMA is suggestive of impaired intracellular Vitamin B12 function and regarded as the most specific marker for Vitamin B12 deficiency with levels above 260–350 nmol/L suggestive of Vitamin B12 deficiency (16). Renal insufficiency may independently elevate MMA and should be considered during interpretation. Homocysteine levels are elevated in both Vitamin B12 and folate deficiency apart from other causes such as hypothyroidism, renal disease, vitamin B6 deficiency, and inherited metabolic disorders. Concurrent elevation of MMA and homocysteine substantially increases diagnostic confidence and is particularly useful when serum vitamin B12 concentrations are borderline or when clinical findings are discordant with laboratory results (7).

Holotranscobalamin (HoloTC), the biologically active fraction of vitamin B12, may detect deficiency earlier than serum vitamin B12 (levels below 35 pmol/L suggest deficiency). However, its use is limited by higher cost, restricted availability, and relatively limited pediatric data (16, 21).

For community-based surveys and epidemiological studies, biomarker selection must balance diagnostic accuracy with feasibility, cost, and laboratory infrastructure. Serum vitamin B12 remains the most commonly used biomarker in community surveys because it is inexpensive, standardized, and widely available. Both the Indian Academy of Pediatrics (IAP) and British guidelines advocate the use of at least two complementary parameters for accurate assessment of vitamin B12 status, particularly in research settings and prevalence studies (21, 26). The most commonly recommended combinations are serum vitamin B12 with HoloTC, or serum vitamin B12 with MMA and/or homocysteine (7, 21, 26). Such combined approaches improve identification of subclinical and functional deficiency, reduce misclassification, and provide a more reliable estimate of population burden.

When biochemical markers confirm Vitamin B12 deficiency but there is suspicion of an inherited or metabolic cause, urine for proteinuria, plasma acylcarnitine profile, urinary organic acids, plasma amino acids should be done as screening and diagnosis can be confirmed by genetic testing (next generation sequencing/whole exome sequencing) (16). *Emerging tests* like CobaSorb assay may help assess intestinal vitamin B12 absorption and identify patients suitable for oral replacement therapy (7, 27).

Anti-intrinsic factor antibody and anti-parietal cell antibody testing should be reserved for children with unexplained, recurrent, or persistent deficiency or when autoimmune gastritis/pernicious anemia is clinically suspected or in presence of other autoimmune disorders like Addisons disease, Type 1 diabetes mellitus etc. (21, 26, 28). Anti-intrinsic factor antibodies are preferred because of their high specificity (21, 26, 28). Anti-parietal cell antibodies, although more sensitive, have lower specificity and may be positive in other autoimmune disorders or occasionally in healthy individuals, therefore best interpreted as supportive evidence rather than as a stand-alone diagnostic test (21, 28).

Bone marrow examination is rarely required and should be reserved for atypical presentations or when alternative diagnoses such as leukemia, aplastic anemia, or myelodysplastic syndrome are being considered (7, 21). Typical marrow findings include hypercellularity, megaloblastic erythropoiesis, giant metamyelocytes, and reversal of the normal myeloid-to-erythroid ratio due to erythroid hyperplasia (22).

### 2.5 Treatment protocol

Vitamin B12 replacement should be initiated promptly when clinical features, dietary history, and laboratory findings suggest deficiency, particularly in the presence of macrocytic anemia or neurological manifestations. If serum vitamin B12 estimation is unavailable, empirical treatment with vitamin B12 should not be delayed. Folic acid should not be administered alone until vitamin B12 deficiency has been excluded, as isolated folate therapy may worsen neurological injury.

#### 2.5.1 Route of administration

##### 2.5.1.1 Oral therapy

High-dose oral vitamin B12 is now recognized as an effective alternative to intramuscular (IM) therapy for many patients with vitamin B12 deficiency. Approximately 1% of a large oral dose (1000–2000 μg/day) is absorbed by passive diffusion independent of intrinsic factor, allowing effective treatment even in patients with pernicious anemia and other malabsorptive disorders (2) as also supported by randomized trials and systematic reviews where it was found to be comparable to IM therapy in normalizing serum vitamin B12 levels and correcting hematological abnormalities (29, 30).

Limited pediatric studies have also demonstrated successful correction of nutritional vitamin B12 deficiency with oral supplementation, although evidence is less robust than in adults (31, 32).

Current evidence supports oral vitamin B12 (1000–2000 μg/day) as first-line therapy for uncomplicated nutritional deficiency and as a maintenance option in compliant patients with pernicious anemia (2, 26). Intramuscular therapy remains preferable in patients with severe neurological manifestations, severe symptomatic anemia, or when rapid correction and assured compliance are required.

In India, where nutritional deficiency is the predominant cause of vitamin B12 deficiency, oral therapy offers practical advantages including lower cost, ease of administration, and improved adherence (33), as also recommended by IAP (21). The suggested daily is dose is 500 μg/day for infants and 1,000 μg/day for older children. It can be given daily for 3 months or tapered (daily → alternate days → twice weekly → weekly → fortnightly → monthly) over at least 3 months.

*Parenteral therapy* is indicated for neurological manifestations, severe anemia or pancytopenia, malabsorption syndromes, inherited disorders of vitamin B12 metabolism, and poor compliance with oral therapy (21).

The regimen recommended by IAP is 25 μg IM/deep SC/IV daily for 2–3 days (especially in malnourished or severely anemic children) then 100 μg daily (50 μg in infants) for 7 days (extend to 3 weeks if neurological disease) followed by 100 μg on alternate days for 7 days then 1,000 μg weekly for 1 month and maintenance of 1,000 μg monthly, orally or parenterally depending on the underlying cause (21).

A lower starting dose of 25–50 μg/day is preferred especially in younger children who are malnourished or severely anemic to prevent life-threatening hypokalemia and in children with neurological symptoms to prevent the worsening of tremors (21, 34, 35).

The IV route is preferred in severe thrombocytopenia to avoid injection-site hematoma (21).

##### 2.5.1.2 Alternative routes: current evidence

###### 2.5.1.2.1 Intranasal vitamin B12

Intranasal hydroxocobalamin bypasses gastrointestinal absorption and offers a painless alternative for children requiring long-term replacement. An adult study as well as a pediatric case series involving 10 children demonstrated normalization of serum vitamin B12 concentrations without reported adverse effects after intranasal hydroxocobalamin therapy (36). However, current pediatric guidelines do not recommend routine use because robust randomized controlled trials are lacking.

Clinical implication: Intranasal therapy appears promising as maintenance therapy in selected compliant patients but should not replace parenteral treatment in severe deficiency or neurological disease.

###### 2.5.1.2.2 Subcutaneous vitamin B12

Deep subcutaneous administration provides pharmacokinetic efficacy similar to intramuscular injection while avoiding muscle trauma and may be preferred in children requiring repeated injections (1). Several metabolic centers successfully use subcutaneous hydroxocobalamin in inherited cobalamin disorders and chronic replacement therapy, particularly when home administration is desirable (27, 46). Available evidence suggests comparable biochemical control to IM therapy, although high-quality pediatric comparative studies remain limited.

Current consensus considers SC administration an acceptable alternative to IM, particularly for long-term treatment (1, 7, 21).

###### 2.5.1.2.3 Sublingual vitamin B12

Sublingual administration bypasses swallowing difficulties and has theoretical advantages through transmucosal absorption. Small pediatric studies and case series have demonstrated correction of hematological abnormalities and normalization of vitamin B12 concentrations using sublingual methylcobalamin or cyanocobalamin (37, 38). Nevertheless, available evidence suggests no clear superiority over conventional oral therapy.

#### 2.5.2 Duration of vitamin B12 therapy

In case of only hematological disease, minimum 3 months therapy is required whereas atleast 6 months of therapy is needed for neurological manifestations (7, 21).

Disorders with irreversible malabsorption, pernicious anemia, inherited disorders, or persistent dietary risk (e.g., strict veganism) may require lifelong replacement therapy (7, 21).

#### 2.5.3 Timeline for recovery of biochemical, hematological and neurological parameters

The recovery of hematological, neurological and biochemical paramareters is as follows (22, 39):

Day 1: Ineffective hematopoiesis reverses, and patients often report improved well-being.

Day 2: Erythropoiesis becomes normoblastic, reticulocyte counts increase, and WBC/platelet counts start to improve.

Day 5: Reticulocyte count peaks, MCV may initially rise as bone marrow responds, and WBC/platelets normalize.

Day 7: MMA and Hcy improves in a week.

Week 2: Neutrophil hypersegmentation and giant meta- myelocytes disappear from the bone marrow, and MCV improves.

Weeks 6–8: Blood counts and RBC indices fully normalize.

Week 6–3 months: Neurological symptoms improve.

Routine monitoring generally consists of clinical parameters, CBC with reticulocyte count at 1 week and CBC at 6–8 weeks (7, 21).

#### 2.5.4 Formulations available

Currently four formulations of Vitamin B12 are available- Cyanocobalamin (CN-Cbl), Hydroxocobalamin (OH-Cbl), Methylcobalamin (Me-Cbl), Adenosylcobalamin (Ado-Cbl) (7, 21).

Cyanocobalamin (CN-Cbl) is the standard synthetic preparation with extensive evidence supporting efficacy.

Hydroxocobalamin (OH-Cbl) has longer tissue retention and requires less frequent injections, making it preferable in pernicious anemia and inherited metabolic disorders (5).

Methylcobalamin (Me-Cbl) although commonly prescribed, has not demonstrated superior hematological or neurological outcomes compared with cyanocobalamin or hydroxocobalamin. It remains an acceptable option because of widespread availability.

All administered forms are ultimately converted intracellularly to active coenzyme forms (7, 21).

#### 2.5.5 Brief approach to treatment of inherited vitamin B12 deficiency

Inherited disorders include intrinsic factor deficiency, Imerslund–Gräsbeck syndrome, transcobalamin II deficiency, and intracellular cobalamin metabolism defects (e.g., cblC) (5).

Lifelong replacement therapy is usually required. Intramuscular hydroxocobalamin is generally preferred, particularly for re-methylation disorders, because of superior intracellular retention. Typical regimens used for cblC disease, Intrinsic factor deficiency and Imerslund–Gräsbeck syndrome include 1 mg IM daily initially, followed by individualized spacing based on metabolic response (weekly, monthly, or less frequently in selected disorders) (5).

Transcobalamin II deficiency usually requires 1 mg IM weekly lifelong (5).

Although high-dose oral therapy has been explored in selected inherited disorders, parenteral therapy remains the standard of care because of more reliable correction and metabolic control (5).

### 2.6 Prevention and public health implications

#### 2.6.1 The problem

Vitamin B12 deficiency is an underrecognized but significant global public health problem, particularly affecting pregnant women, infants, and populations with limited consumption of animal-source foods (44, 45). Maternal vitamin B12 status is the principal determinant of fetal stores and breast milk cobalamin concentrations. Consequently, infants born to deficient mothers have reduced hepatic reserves and are at high risk of developing deficiency during exclusive breastfeed- ing, particularly after 4–6 months of age (12). In a Cochrane review of studies from LMICs, 26–51% of pregnant women were vitamin B12 deficient and 30–46% were anemic, emphasizing the magnitude of this preventable nutritional disorder (40). The burden is driven by maternal undernutrition, food insecurity, vegetarian or vegan dietary practices, malabsorption disorders, and limited access to antenatal nutritional care.

Low infant cobalamin levels have been associated with impaired motor development and adverse neurodevelopmental outcomes (12, 13). Because neurological injury occurring during infancy may be only partially reversible despite treatment, prevention before and during pregnancy is of paramount importance (12, 13, 33).

#### 2.6.2 Gaps

Despite its clinical importance, there is currently no universally implemented global program dedicated specifically to prevention of vitamin B12 deficiency during pregnancy. Unlike iron and folic acid supplementation, vitamin B12 is generally provided through multiple micronutrient supplements or targeted nutritional interventions rather than routine antenatal care policies (45). Nevertheless, several countries and professional organizations advocate vitamin B12 supplementation or fortified foods for women at increased risk, particularly those consuming vegetarian or vegan diets (41, 42).

#### 2.6.3 Way ahead

- Screening strategies to focus on high-risk populations, including women with vegetarian or vegan dietary patterns, malnutrition, previous bariatric surgery, gastrointestinal malabsorption, pernicious anemia, or unexplained macrocytic anemia.
- Assessment should include a detailed dietary history, complete blood count with reticulocyte indices, and serum vitamin B12 measurement where available. Newer markers such as reticulocyte hemoglobin and immature reticulocyte fraction may facilitate earlier diagnosis, although their availability remains limited.
- Integration of vitamin B12 into existing antenatal care services, iron–folic acid programs, and maternal nutrition initiatives offers an opportunity to improve coverage without substantial additional infrastructure.
- Fortification of staple foods and plant-based alternatives with vitamin B12 is another population-level intervention which could improve intake across vulnerable populations and reduce health disparities, although further implementation research is required to determine optimal delivery strategies.
- Public health education should complement these measures by promoting awareness of vitamin B12-rich and fortified foods, culturally acceptable dietary sources, appropriate complementary feeding practices, recognition of deficiency symptoms, and the importance of maternal nutrition before conception, during pregnancy, and throughout lactation.

Together, maternal screening, targeted supplementation, food fortification, and nutrition education provide a comprehensive strategy to reduce the global burden of vitamin B12 deficiency and prevent irreversible neurodevelopmental impairment in children.

## 3 Conclusion

Vitamin B12 deficiency in children is a common but frequently underdiagnosed condition with potentially serious hematological and neurological consequences. Clinical manifestations range from isolated anemia to developmental regression and metabolic disease. Early recognition and prompt treatment are essential to prevent irreversible neurological injury. Functional biomarkers and molecular diagnostics have improved identification of inherited cobalamin disorders, which require specialized and lifelong management. Increased awareness, nutritional interventions, and early screening strategies are critical for improving pediatric outcomes worldwide.
